# Ratio of water to cement and supplementary cementitious materials on mechanical and impact resistance properties of reactive powder concrete

**DOI:** 10.1038/s41598-025-92097-3

**Published:** 2025-03-03

**Authors:** Haoyu Tan, Peilong Yuan, Dazhao Sun, Xiang Fan, Cheng Wang, Junrong Liu

**Affiliations:** 1 CCCC-SHEC Dongmeng Engineering Co., Ltd, Xi’an, 710076 China; 2 School of Highway, Chang’an University, Xi’an, 710064 China

**Keywords:** Reactive powder concrete, Supplementary cementitious materials, Mechanical properties, Impact resistance, Engineering, Materials science

## Abstract

Reactive powder concrete (RPC) is a novel high-performance building material widely used in large-scale engineering structures due to its superior mechanical properties and durability. However, structural failure can still occur under dynamic load impacts. Therefore, optimizing the mechanical properties and impact resistance of RPC remains a critical issue for enhancing its engineering applications. In this study, the mechanical properties and impact resistance of RPC were investigated by adjusting the water-cement ratio and incorporating supplementary cementitious materials (SCMs), such as fly ash microspheres (FAM) and silica fume (SF). The effects of these adjustments on water absorption, strength, and impact resistance were assessed. Three water-cement ratios (0.16, 0.18, and 0.20) and various proportions of FAM and SF were selected to evaluate water absorption, compressive strength, bending strength, and impact resistance. The results indicated that reducing the water-cement ratio enhanced the densification of the concrete, reduced water absorption, and improved both compressive and bending strength. Specifically, when the water-cement ratio was 0.16 and FAM and SF were synergistically incorporated, the compressive strength reached 134.4 MPa, the bending strength reached 16.86 MPa, and the impact resistance was 22,838.4 J. Impact test results revealed that combining a low water-cement ratio with an appropriate amount of SCMs effectively increased energy absorption capacity and significantly slowed crack propagation. Analysis based on the Weibull distribution model demonstrated a more pronounced probability distribution of the number of impacts, suggesting that the optimization measures improved the impact resistance of RPC.

## Introduction

Large engineering structures may experience significant dynamic load impacts from force majeure events, such as rockfalls, vehicle and ship collisions, earthquakes, or terrorist attacks, during operation^1,2^. Ordinary concrete, the most widely used construction material in engineering structures, offers advantages such as excellent bearing performance, abundant raw material sources, and moderate cost. However, due to the presence of large aggregates and the lack of flexible reinforcing materials, its toughness is limited, making it brittle and prone to cracking^3,4^. This susceptibility can lead to structural failure under strong dynamic loads, posing a significant risk to the stability of engineering structures and the safety of people and property. RPC developed based on the theory of closest particle packing by Richard and Cheyrezy at Bouygues Laboratory in France in 1990^5^, is an advanced cementitious composite material. RPC is typically composed of a mixture of cement, SF, quartz sand, and other fine powders, along with water and high-efficiency water-reducing agents. It employs a low water-cement ratio and particles with sizes smaller than 600 μm^6^. RPC offers advantages such as high densification, ultrahigh strength, high toughness, and exceptional durability, which surpass those of ordinary concrete. It is applicable to large-scale engineering structures, including bridges, tunnels, underground caverns, and prefabricated structures^7–9^.

Recent studies on RPC have provided valuable insights, but further improvements are necessary to enhance its strength and toughness. To optimize the quasi-brittle confinement of RPC, increase its toughness, and reduce the maintenance costs of engineered structures, flexible fibre materials are commonly used for reinforcement. Commonly used flexible fibre materials include steel fibres^10^, polypropylene fibres^11^, and basalt fibres^12^. The incorporation of steel fibres significantly improves the compressive strength, tensile strength, bending strength, and toughness of RPC^13–15^. In 1995, Cheyrezy et al.^16^ incorporated steel fibres into RPC, effectively increasing its strength and toughness. Al-Hassani et al.^17^ reported that the addition of 3% steel fibres by volume increased the tensile and bending strengths of RPC by 258.4% and 217.3%, respectively. GE Lincai et al.^18^ reported that the incorporation of 1.65% ultrafine steel fibres into RPC resulted in a 32.22% increase in compressive strength and a 211.29% increase in splitting tensile strength under standard curing conditions. The excellent performance of steel fibres effectively mitigates the impact of macrocracking on the mechanical properties of RPC, making them a widely used material for enhancing the mechanical properties of RPC.

For every tonne of cement produced, approximately one tonne of CO_2_ is released, contributing 8–9% of global carbon emissions and 2–3% of energy consumption^19,20^. Researchers have long focused on reducing carbon emissions in cement manufacturing or finding alternative SCMs to achieve carbon neutrality in the industry. The incorporation of SCMs, such as fly ash, SF, and blast furnace slag^21^, to partially replace cement enhances concrete performance by reducing pore size, lowering the heat of hydration, increasing strength and durability, enhancing impermeability, and effectively reducing cement usage^22^. Fly ash, a byproduct of coal combustion in power plants, consists of fine spherical particles^23^. It can replace part of the cement and participate in a volcanic ash reaction with calcium hydroxide to produce calcium silicate hydrate (C-S-H) gel, the primary product of SCMs^24^. The fineness of fly ash is a key factor influencing the volcanic ash reaction^25^. By extracting spherical particles from fly ash through systematic separation and impurity removal, FAM are produced, providing tighter filling of pores in concrete and enhancing strength and durability^26^. The introduction of SF, with a high surface area to volume ratio, results in a significant reduction in the total pore volume of RPC cement paste, generating additional calcium silicate hydrate and accelerating the reaction with Ca(OH)_2_^27^, thus forming a dense microstructure that imparts high durability and low permeability to RPC. This makes RPC a promising material for use in specialized pre-stressing and prefabricated components^28^.

Although numerous studies have focused on enhancing the mechanical properties of RPC by using fibers or SCMs, these studies often fail to reveal the strength growth patterns and toughness characteristics associated with these materials. In this study, the mechanical properties of RPC were enhanced by adjusting the water-cement ratio and incorporating SCMs (FAM and SF). This research provides a detailed evaluation of the effects of these materials on water absorption, compressive and bending properties at different curing ages, as well as on low-velocity impact resistance and toughness.

## Test materials and methods

### Test materials

**Fig. 1:**
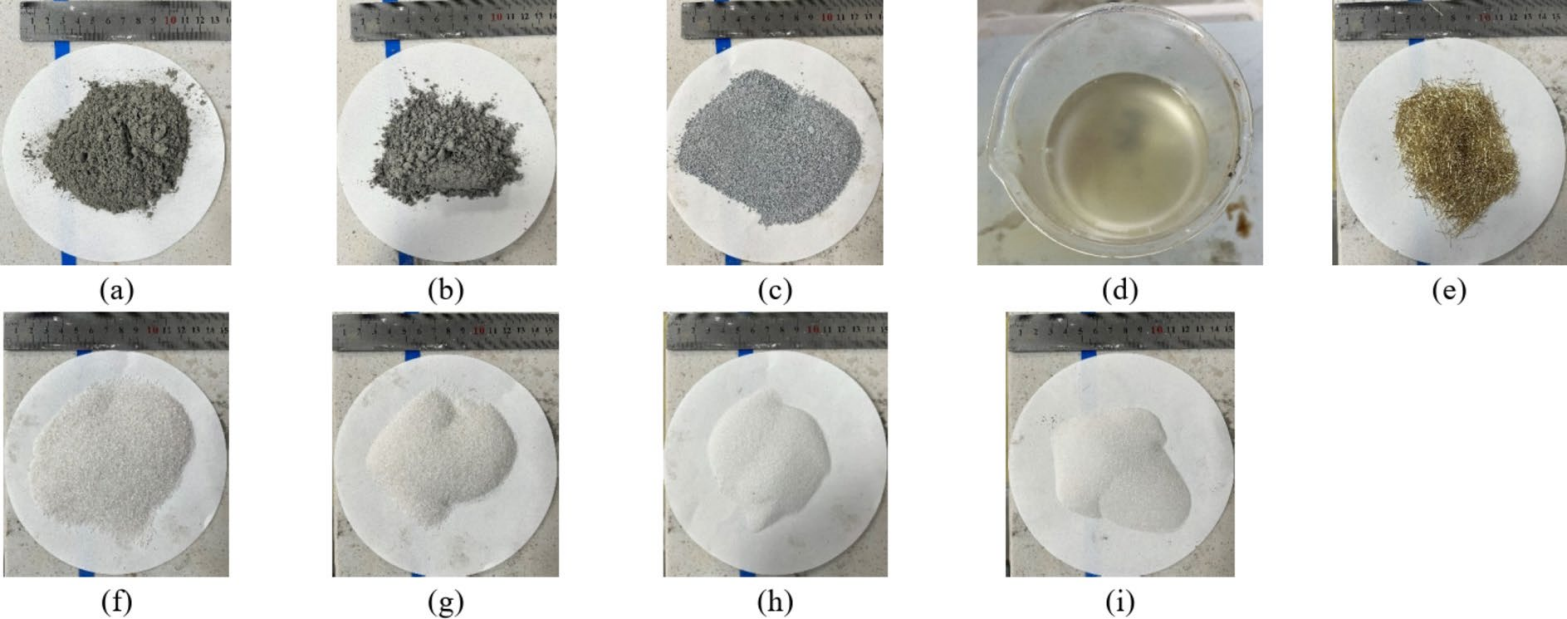
Test materials, (**a**) P·O 52.5 (**b**) FAM (**c**) Highly active SF (**d**) Efficient water-reducing agent (**e**) Microfine copper-plated steel fibres (**f**) Coarse-grained quartz sand (**g**) Medium-grained quartz sand (**h**) Fine-grained quartz sand (**i**) Ultrafine-grained size quartz sand.

The technical specifications for RPC used in this study followed GB/T 31,387 − 2015 “Reactive Powder Concrete”^29^, and the materials utilized are presented in Fig. 1. P·O 52.5 ordinary Portland cement (Fig. 1a) was used as the primary cementitious material, while FAM and highly active micro-SF were employed as supplementary materials (Fig. 1b, c)). The specific parameters of these materials are detailed in Table 1. A high-performance polyhydroxy acid water-reducing agent was used, with a maximum water-reducing rate of 45% (Fig. 1d). Microfine copper-plated steel fibers with a diameter of 0.18 mm, a length of 12 mm (Fig. 1e), and a tensile strength greater than 3000 MPa were selected. To achieve high compactness, a homogeneous structure, and minimal porosity, fine aggregates were selected from quartz sand with SiO_2_ content greater than 97%. The quartz sand was classified into four particle sizes: coarse (1.0–0.63 mm), medium (0.63–0.315 mm), fine (0.315–0.16 mm), and ultrafine (< 0.16 mm) (Fig. 1f, i)^30^.

**Table 1:** Parameters of the cementitious materials.

Parameter	*P*·O 52.5	FAM	SF
Fineness (sieve residue)	2.8%	0.6%	3%
Loss on burning	2.37%	0.7%	3.42%
SO_3_	2.18%	0.5%	0.8%
MgO	1.4%	1.51%	1.8%
Specific surface area	348 m^2^/kg	233 m^2^/kg	191 m^2^/kg
Cl	0.024%	0.015%	0.07%
SiO_2_	24%	56%	92.3%
Moisture content	0.06%	0.05%	0.09%
CaO	63.2%	9%	0.8%
Density	3.1 g/cm^3^	2.0 g/cm^3^	2.3 g/cm^3^

### Mixing ratio design

The reference mixture design for RPC^31^ followed the proportions cement: SCMs: fine sand: water = 1:0.25:1.1:0.2, with 1.5% microfine copper-plated steel fibres by volume and 1% high-performance polyhydroxy acid water-reducing agent by cementitious material mass. The water-cement ratio, FAM content, and SF dosage were varied as key parameters. Three water-cement ratios, 0.16, 0.18, and 0.20, were designed, along with various proportions of FAM and SF in the total SCMs. The specific mixture designs are shown in Table 2. For example, the label “0.16-F0-S1” represents a mixture with a water-cement ratio of 0.16, F0 indicates that the proportion of FAM in the SCMs is 0%, and S1 indicates that the proportion of SF is 100%.

**Table 2:** Design of the RPC proportions.

Test group number	Specimen number	Water/cement	cement	FAM	SF	Quartz sand				Water	Water reducing agent	Micro steel fibers
						1 ~ 0.63	0.63 ~ 0.315	0.315 ~ 0.16	0.16			
0.2-F0-S0	a1	0.2	1.25	0	0	0.495	0.33	0.22	0.055	0.2	0.06	1.50%
0.18-F0-S1	a2	0.18	1	0	0.25	0.495	0.33	0.22	0.055	0.225	0.06	1.50%
0.18-F0.5-S0.5	a3	0.18	1	0.125	0.125	0.495	0.33	0.22	0.055	0.225	0.06	1.50%
0.18-F1-S0	a4	0.18	1	0.25	0	0.495	0.33	0.22	0.055	0.225	0.06	1.50%
0.16-F0-S1	a5	0.16	1	0	0.25	0.495	0.33	0.22	0.055	0.2	0.06	1.50%
0.16-F0.5-S0.5	a6	0.16	1	0.125	0.125	0.495	0.33	0.22	0.055	0.2	0.06	1.50%
0.16-F1-S0	a7	0.16	1	0.25	0	0.495	0.33	0.22	0.055	0.2	0.06	1.50%

To achieve thorough mixing of the RPC, a 60 L forced mixer was employed. Initially, the weighed quartz sand, microfine copper-plated steel fibres, cement, SCMs, and other dry ingredients were pre-mixed for 4 min to achieve initial fiber dispersion. Once the mixture reached homogeneity, a high-performance polyhydroxy acid water-reducing agent (with 45% maximum water-reducing rate) and water were added to enhance workability and further promote fiber distribution, followed by an additional 4 min of mixing to ensure even fiber dispersion and prevent the agglomeration of steel fibres. This two-stage mixing protocol combined with high-efficiency water reducer effectively maintained fiber dispersion homogeneity. The resulting mixture was then poured into moulds and subjected to vibration to achieve densification. After 24 h of natural curing indoors at a temperature of 20 ± 2 °C, the specimens were carefully demoulded and transferred to a standard constant-temperature and -humidity curing chamber maintained at 20 ± 2 °C and 95% relative humidity, in compliance with GB/T 31,387 − 2015 standard for RPC. These controlled conditions were implemented to ensure uniform curing, consistent development of concrete properties, and reliable simulation of typical engineering conditions. The specimens remained in this environment until the specified curing age was attained. Subsequently, mechanical tests were conducted on the specimens^32,33^.

### Test methods

#### Absorbency

The specimens were immersed in water at 20 ± 2 °C for 28 days. A 10 mm-diameter rebar cushion was placed at the lower part of the specimen, with the water surface 25 mm above the top surface. After 24 h of immersion, the specimens were removed, the surface water was wiped off, and the mass was recorded. The specimens were then reimmersed for another 24 h, after which the mass change was measured. Immersion continued until the change in mass between two consecutive 24-hour intervals was less than 0.2%. At that point, the immersion was stopped, and the final mass was recorded as the surface-dry mass $\:{m}_{s}$ of the water-saturated specimen.

The specimens were subsequently placed in a blast drying oven at 105 ± 5 °C for 24 h, cooled to room temperature and weighed. Drying was continued for another 24 h, with mass measurements taken until the change in mass between two consecutive 24-hour intervals was less than 0.2%. At that point, drying was stopped, and the final mass was recorded as the dried specimen mass $\:{m}_{d}$^34^.

The water absorption rate was calculated according to Eq. (1):

1 $$\:{\varvec{W}}_{\varvec{a}}=\frac{{\varvec{m}}_{\varvec{s}}-{\varvec{m}}_{\varvec{d}}}{{\varvec{m}}_{\varvec{d}}}\times\:100\varvec{\%}$$

where $\:{W}_{a}$ is the water absorption rate of RPC (%), $\:{m}_{s}$ is the surface-dry mass of the saturated specimen (g), and $\:{m}_{d}$ is the mass of the dried specimen (g).

#### Uniaxial compression test

**Fig. 2:**
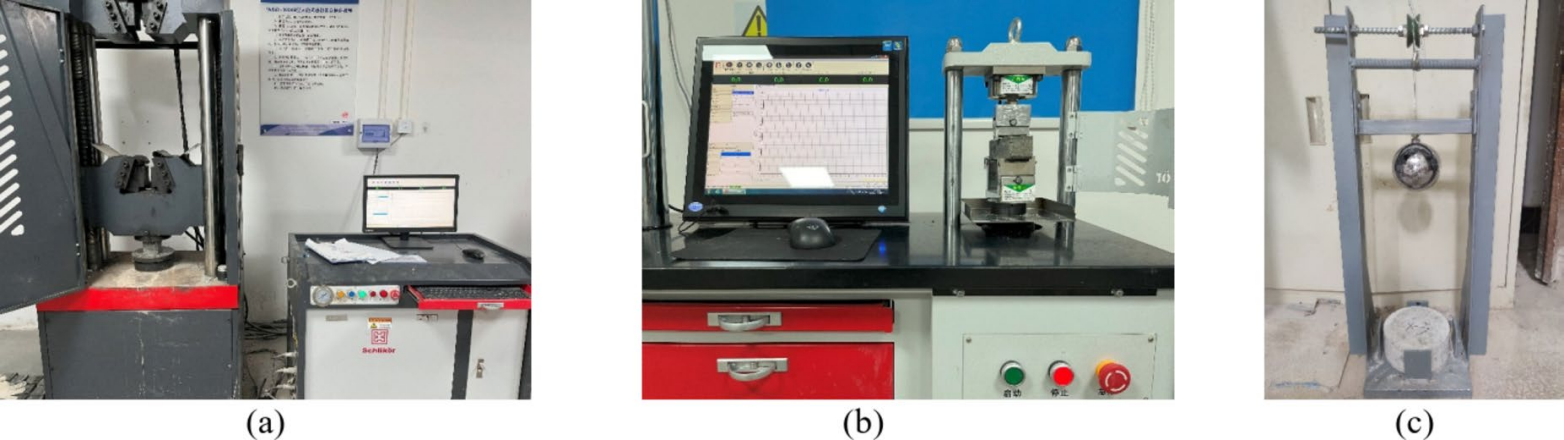
Test setup. (**a**) Uniaxial compression equipment (**b**) Three-point bending equipment (**c**) Homemade impact test equipment.

According to the specification JGJ/T70-2009^35^, uniaxial compression tests were conducted on cubic specimens with a side length of 70.7 mm at 3, 7, and 28 days using a WAW-1000B universal testing machine (Fig. 2a). The compressive properties of RPC at different ages were determined, and the compressive strength was calculated by averaging the results from four specimens.

#### Three-point bending test

A three-point bending test was performed using a YAW-SERIES fully automatic cement bending and compressive integrated machine to measure the bending strength (Fig. 2b). The specimens had cross-sectional dimensions of 40 mm × 40 mm and a total length of 160 mm. The support span in the bending test was 100 mm, and the displacement rate was set at 0.05 mm/s. The specimens were tested at 3, 7, and 28 days, and the bending strength was calculated by averaging the values from four specimens of each mixture.

The bending strength was estimated from the load–deflection response shown in Eq. (2).

2 $$\:{\varvec{\sigma\:}}_{\varvec{F}}=\frac{{\varvec{P}}_{\varvec{F}}\varvec{L}}{{\varvec{b}\varvec{d}}^{2}}$$

where $\:{\varvec{\sigma\:}}_{\varvec{F}}$ is the bending strength (MPa), $\:{\varvec{P}}_{\varvec{F}}$ is the maximum bending load applied (N), and b, d, and L are the width, depth, and span length (mm) of the specimen, respectively.

#### Falling hammer impact test

Given that repeated traffic-induced live loads can compromise the durability of concrete structures, impact resistance tests were conducted on RPC. Following the CECS 13:2009 Standard for Test Methods for Fiber Concrete^36^, a custom-built impact resistance device with a rigid horizontal base plate was utilized, as illustrated in Fig. 2c. The test specimens, measuring 150 mm in diameter and 65 mm in height, were cured for 28 days. A 4.5 kg dense steel ball was positioned on the upper surface of each specimen and allowed to free-fall from a height of 450 mm to induce impact. The number of impacts required to initiate cracking and cause final failure of the specimen was recorded. These measurements were used to evaluate the energy absorption capacity and assess the impact toughness of the material.

The impact energy of RPC was calculated as:

3 $$\:W=mghn$$

where W is the impact work, J; m is the mass of the falling hammer, kg; g is the acceleration of gravity, taken as 9.81 m/s^2^; h is the fall height of the impact hammer; and n is the number of impacts.

## Results and discussion

### Absorbency

**Fig. 3:**
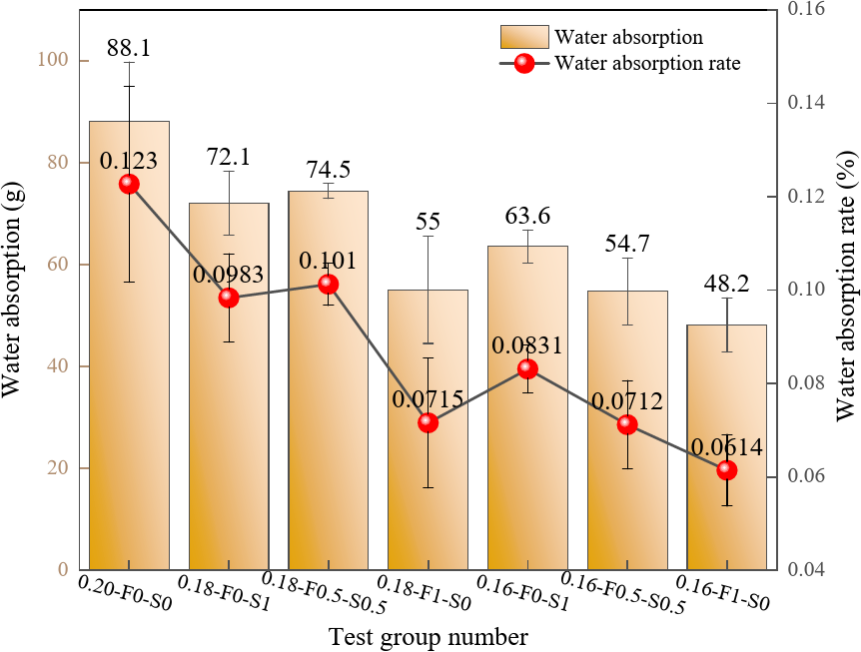
Variations in the water absorption and water absorption rate.

As depicted in Fig. 3, both water absorption and water absorption rates vary significantly across different test groups, influenced by changes in the water-cement ratio and SCMs composition. Generally, a higher water-cement ratio correlates with increased water absorption and absorption rates. For instance, the 0.20-F0-S0 test group, with a water-cement ratio of 0.20, exhibited the highest water absorption of 88.1 g and a water absorption rate of 0.123%. This outcome is attributed to the higher porosity resulting from an elevated water-cement ratio, which facilitates water penetration and increases absorption.

When FAM and SF were combined in equal proportions, as in the 0.16-F0.5-S0.5 group, water absorption decreased to 63.6 g, and the absorption rate dropped to 0.0831%. The use of FAM alone also significantly reduced both water absorption and absorption rates. The 0.16-F1-S0 group demonstrated the lowest water absorption of 48.2 g and an absorption rate of 0.0614%, highlighting the high fineness and reactivity of FAM in reducing porosity and limiting water penetration pathways. Additionally, the incorporation of SF further densified the internal structure of RPC by filling pores and generating additional gelling products through pozzolanic reactions. In summary, the optimization of RPC composition through the reduction of the water-cement ratio and incorporation of SCMs significantly reduces the permeability and increases the durability of RPC.

### Mechanical property analysis

#### Compressive strength

##### Uniaxial compression damage characteristics

**Fig. 4:**
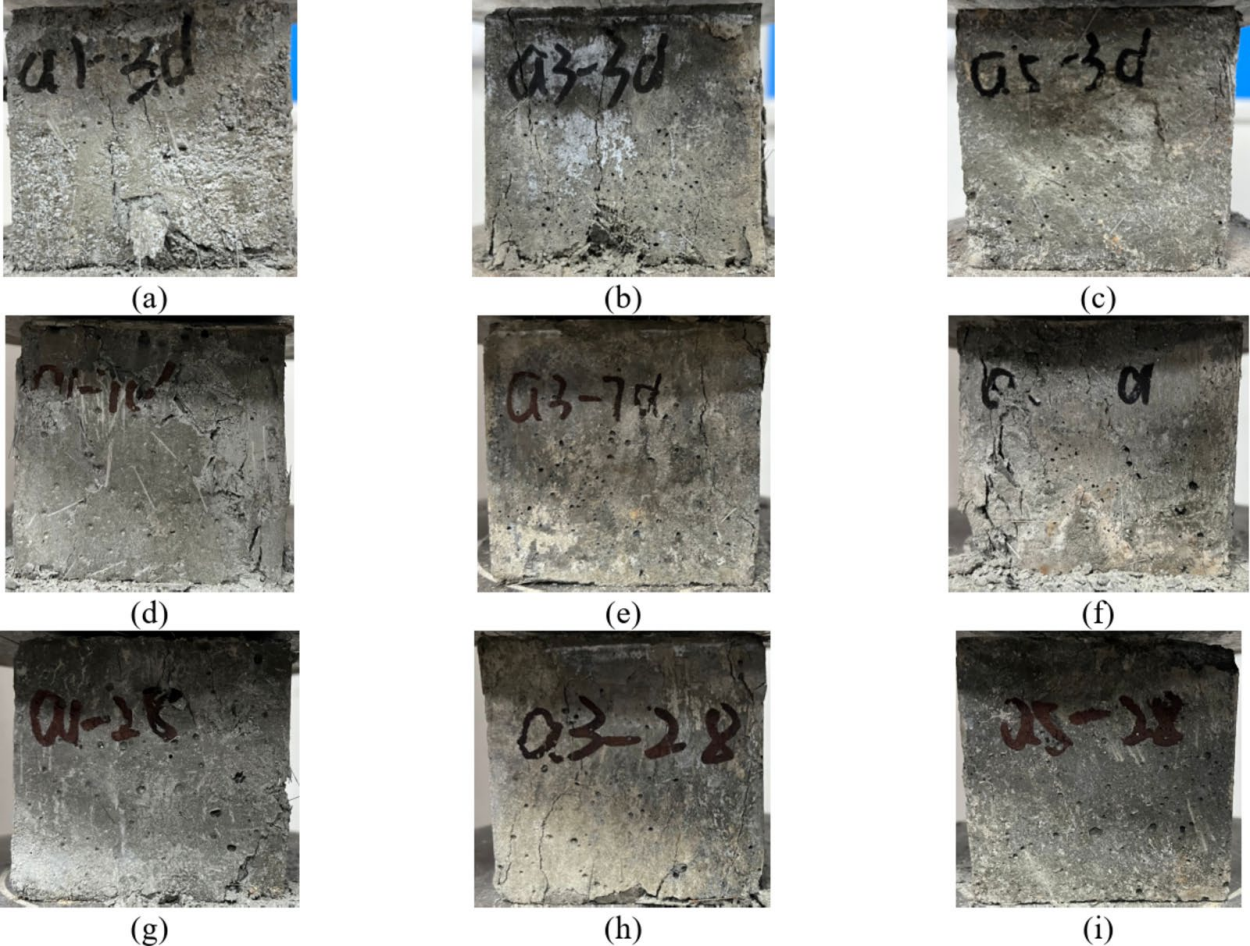
Compressive damage characteristics with age. (**a**) a1-3d (**b**) a3-3d (**c**) a5-3d (**d**) a1-7d (**e**) a3-7d (**f**) a5-7d (**g**) a1-28d (**h**) a3-28d (**i**) a5-28d.

Cracking patterns serve as key indicators of the damage characteristics of RPC, with the water-cement ratio being a critical factor influencing its strength. Lower water-cement ratios typically result in denser, stronger concrete. As shown in Fig. 4, the specimen with a water-cement ratio of 0.2 (Fig. 4a) exhibits more cracks at the same age, with more pronounced damage characteristics. In contrast, the specimen with a water-cement ratio of 0.18 and 0.16 (Fig. 4b, c) exhibits superior compressive properties at an early age, fewer cracks after the peak stress, and more uniform crack propagation during failure. Compared to the group without SCMs admixture (Fig. 4d), the specimens containing FAM and SF (Fig. 4e, f) display more uniform crack distributions and smoother damage characteristics at failure. This behaviour likely occurs because the incorporation of fly ash and SF enhances the densification and bonding of the concrete, which improves its damage resistance. The damage characteristics of the RPC specimens under uniaxial compression significantly vary with age. At early curing ages (3 days), the specimens exhibit more cracks and irregular damage, whereas at later curing ages (28 days), the cracks as shown in Fig. 4g, i) propagate more uniformly, and the damage area increases. This result suggests that as the age of RPC increases, its internal structure stabilizes, and crack development and propagation after the peak stress become more pronounced. This change is attributed to the ongoing internal hydration reactions and microstructural development, which increase the strength and toughness of the concrete with increasing curing age.

##### Effect of age on the compressive strength

**Fig. 5:**
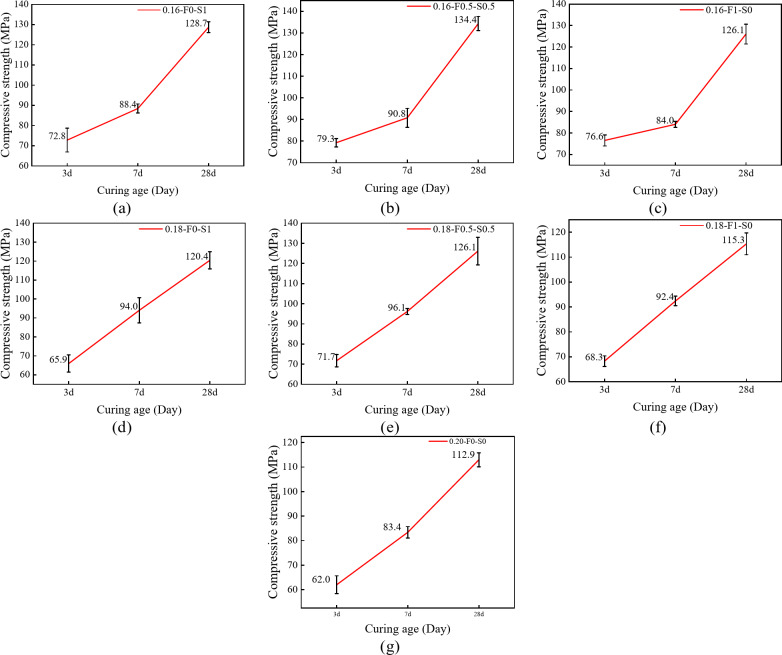
Compressive strength values at different curing ages. (**a**) 0.16-F0-S1 (**b**) 0.16-F0.5-S0.5 (**c**) 0.16-F1-S0 (**d**) 0.18-F0-S1 (**e**) 0.18-F0.5-S0.5 (**f**) 0.18-F1-S0 (**g**) 0.20-F0-S0.

Figure 5 illustrates the compressive strength development of different test groups over curing time. The compressive strength of all groups increased significantly from 3 to 28 days of curing. Early strength gains (3 to 7 days) were rapid, with most specimens achieving or exceeding 85 MPa by 7 days (Fig. 5a, b, and d-f). For example, the 0.18-F0-S1 group exhibited a strength increase from 65.9 MPa at 3 days to 94.0 MPa at 7 days, representing a growth rate of 42.7%. This trend indicates that prolonged curing accelerates hydration reactions, enhancing structural compactness and compressive strength. Strength continued to increase beyond 7 days, with all specimens surpassing 110 MPa by 28 days. For the test group in which one SCM was incorporated alone, FAM promoted early strength growth, as shown in Fig. 5c, f, the 3-day compressive strengths of specimens 0.16-F1-S0 and 0.18-F1-S0 were 76.6 MPa and 68.3 MPa, respectively, which were higher than those of specimens 0.16-F0-S1 and 0.18-F0-S1, which were 3.8 MPa and 2.4 MPa. And SF played more role in the later strength growth with the increase of the age of maintenance. Some mixtures exceeded 130 MPa, such as the 0.16-F0.5-S0.5 (Fig. 5b) group, which increased from 90.8 MPa at 7 days to 134.4 MPa at 28 days, a growth rate of 48%. This sustained strength development suggests ongoing hydration and microstructural refinement. Among all groups, the 0.16-F0.5-S0.5 mixture, incorporating both FAM and SF, achieved the highest strengths, with 3-day and 28-day values of 76.6 MPa and 134.4 MPa, respectively. For the 0.20-F0-S0 group with a high water-cement ratio (Fig. 5g), the 3-day strength was relatively low at 62.0 MPa but increased to 112.9 MPa by 28 days.

##### Effects of the water-cement ratio and SCMs composition on the compressive strength

**Fig. 6:**
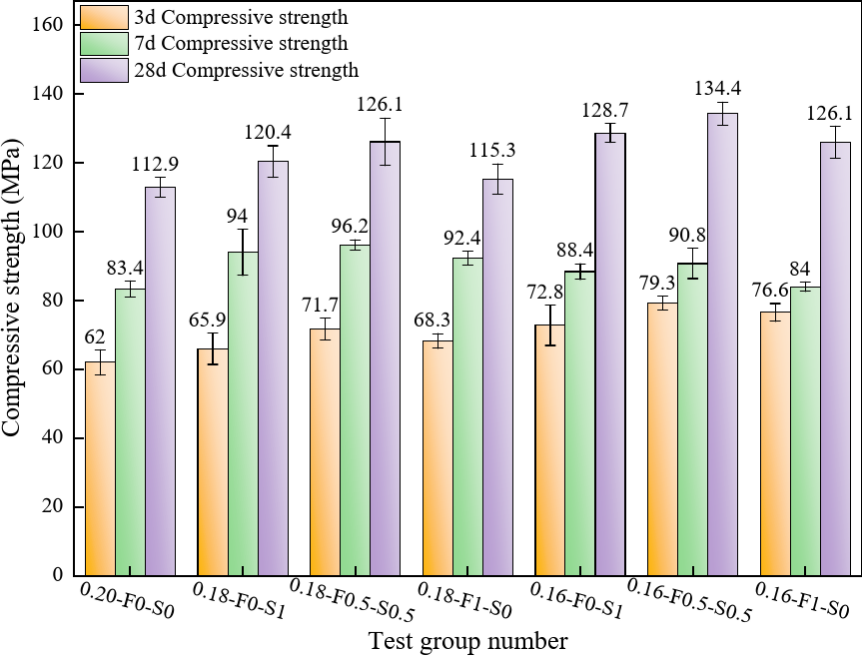
Effects of the water-cement ratio and SCMs composition on the compressive strength.

Figure 6 further demonstrates the effects of varying water-cement ratios and SCMs admixtures on RPC compressive strength. Groups incorporating SF (0.18-F0-S1 and 0.16-F0-S1) consistently exhibited higher strengths at all ages. The compressive strengths at 3, 7, and 28 days increased sequentially, indicating that the pozzolanic reaction of SF enhances strength both in the early stages and over the long term. In contrast, groups incorporating FAM alone showed slightly lower performance. The 0.18-F0.5-S0.5 and 0.16-F0.5-S0.5 groups, with equal proportions of FAM and SF, achieved the highest 28-day strengths of 134 MPa and 126 MPa, respectively. This suggests that the synergistic combination of FAM and SF improves material densification and enhances reactive powder performance through complementary hydration mechanisms, particularly benefiting long-term strength. The 0.20-F0-S0 group, with a water-cement ratio of 0.20, exhibited the lowest compressive strength across all ages, with a 28-day strength of only 112.2 MPa. The high water-cement ratio increased porosity, limiting strength development. This underscores the importance of reducing the water-cement ratio and incorporating SCMs to enhance densification and compressive strength. Overall, the observed strength increases with age highlight the significant influence of the water-cement ratio and SCMs on both early and long-term strength development in RPC.

At an age of 7 days, the compressive strengths of the test groups with a water-cement ratio of 0.16 are generally lower than those of the test groups with a ratio of 0.18. For example, the compressive strength of the 0.16-F0-S1 group at 7 days is 88.4 MPa, whereas that of the 0.18-F0-S1 group is 94 MPa. This phenomenon may be attributed to the fact that, under the conditions of a lower water-cement ratio, there is an overall favourable effect on the densification of the Reactive powdered concrete and its long-term strength. However, a lower water-cement ratio may lead to an insufficient water supply for the hydration reaction at an early age, which slows the formation of hydration products and adversely affects the development of the early strength. Additionally, a lower water-binder ratio may increase the viscosity of the concrete, further hindering the early hydration reaction^37^.

#### Bending strength

##### Characteristics of the three-point bending damage

**Fig. 7:**
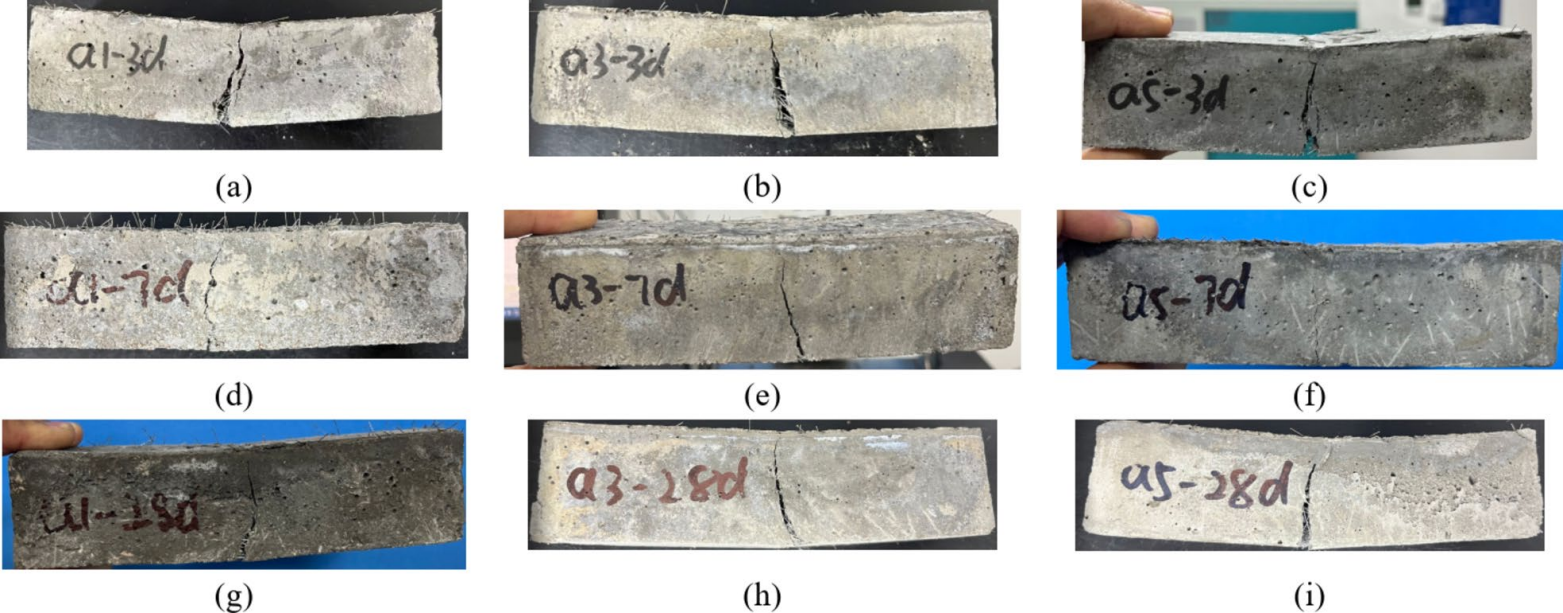
Characteristics of the bending damage with the curing age. (**a**) a1-3d (**b**) a3-3d (**c**) a5-3d (**d**) a1-7d (**e**) a3-7d (**f**) a5-7d (**g**) a1-28d (**h**) a3-28d (**i**) a5-28d.

**Fig. 8:**
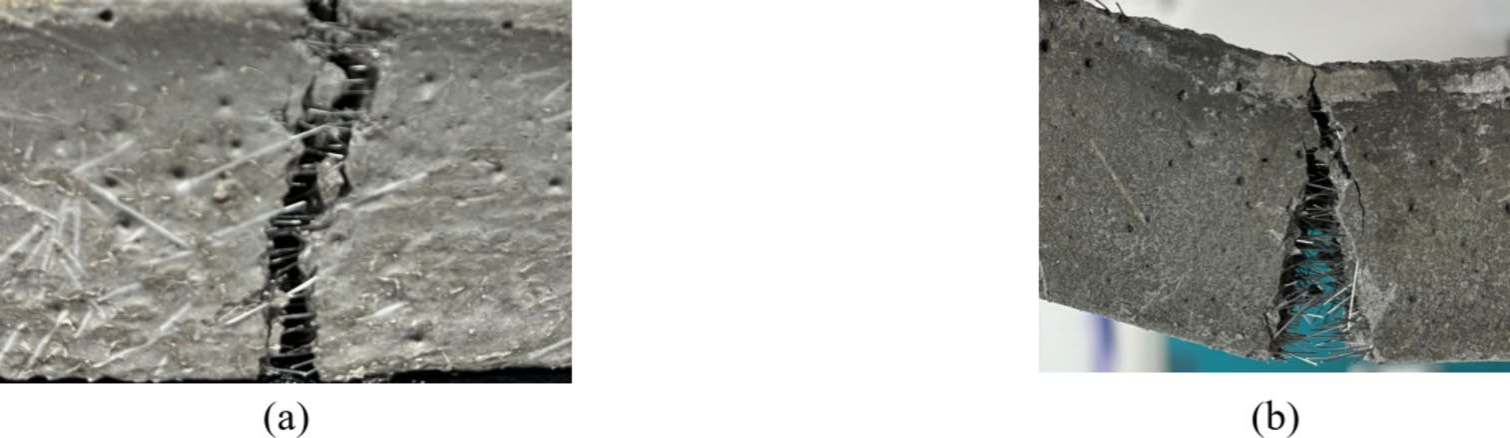
Fibre bridging effect. (**a**) Fibre bridging (**b**) Fibre pullout.

As shown in Fig. 7, during bending failure, fine cracks typically originate from the bottom of the span and gradually expand upwards as the load increases, ultimately leading to fracture of the specimen. The cracking pattern varies across the specimens, likely due to differences in age, water-cement ratio, and SCMs composition. At 3 days of curing, when the specimens reach the peak bending strength, the cracks are more pronounced, and the openings are larger (Fig. 7a-c). This result suggests that a shorter curing period does not allow sufficient internal hydration of the cement, resulting in a weaker bond strength between the internal components. As a result, the cracks are more extensive, and the bending deflections at the peak strength are greater. In this stage, the microfine steel fibres significantly contribute to load-bearing after the peak, providing a clear fibre bridging effect, as illustrated in Fig. 8a.

At 7 days of curing, after the peak bending strength is reached, the crack openings of the specimens are significantly reduced (Fig. 7d-f). This phenomenon can be attributed to the fiber bridging mechanism: as microcracks initiate and propagate, the microfine steel fibers bridge across these cracks, effectively resisting stresses and limiting crack growth through their high tensile strength and elasticity. With the curing time up to 28 days, the typical damage pattern of the specimens did not change under bending load. When the load reached its peak, the specimen gradually cracked from the bottom of the span and extended to the top. In this phase, the cement matrix primarily bears the load after the peak strength, with the microfine steel fibres playing a secondary role (Fig. 7g-i). During the cracking process, obvious fibre pull-out and bridging could be observed. As loading continues, the fiber bridging effect becomes more pronounced, allowing the RPC to absorb additional energy through fiber deformation and pullout mechanisms. The load-bearing contribution of the microfine steel fibres becomes more prominent, effectively dissipating energy and preventing rapid crack widening, eventually leading to their pullout from the cement matrix, at which point the specimens completely fail, as shown in Fig. 8b.

##### Effect of the curing age on the bending strength

**Fig. 9:**
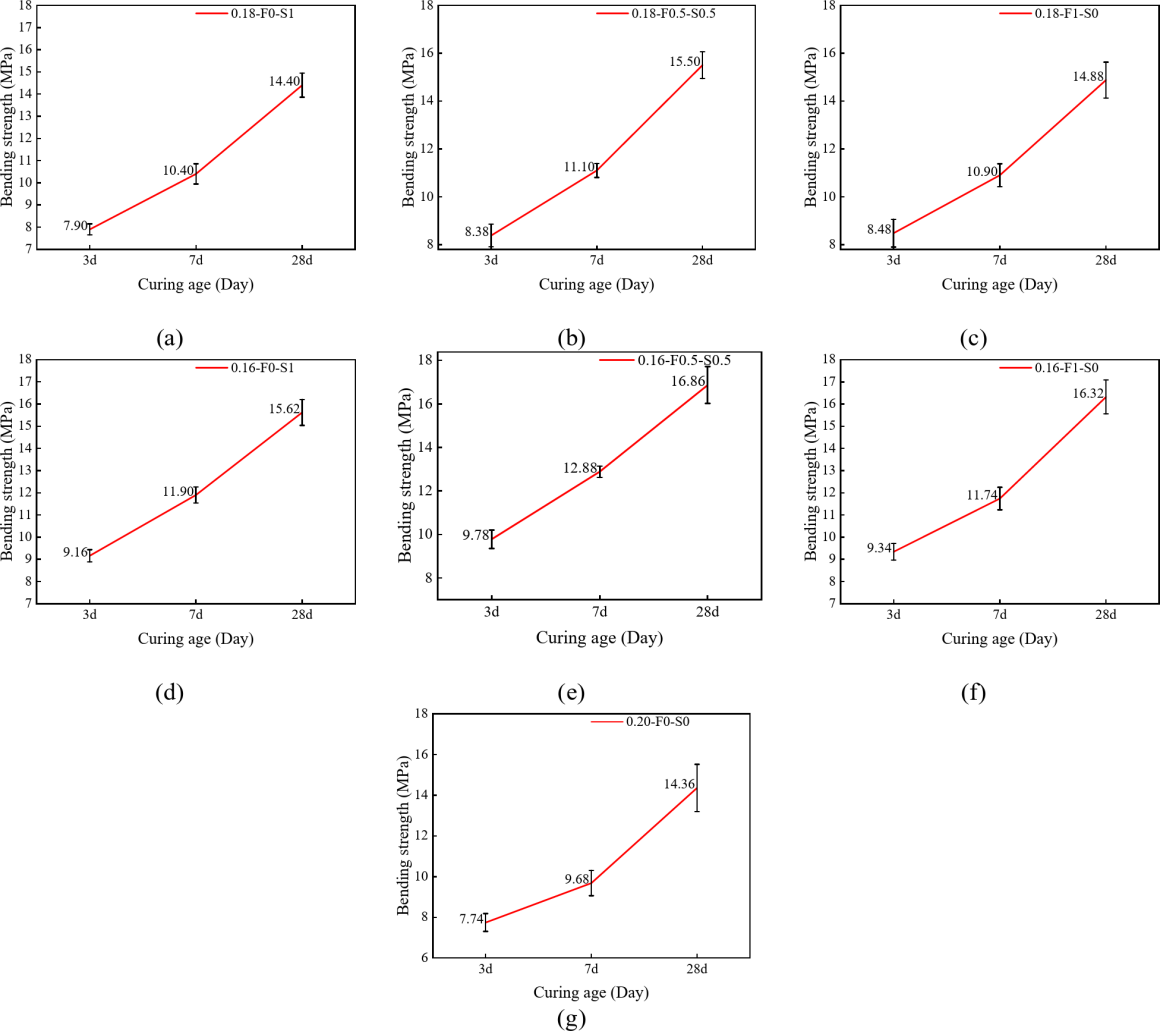
Variation in the bending strength with curing age. (**a**) 0.18-F0-S1 (**b**) 0.18-F0.5-S0.5 (**c**) 0.18-F1-S0 (**d**) 0.16-F0-S1 (**e**) 0.16-F0.5-S0.5 (**f**) 0.16-F1-S0 (**g**) 0.20-F0-S0.

As shown in Fig. 9, the overall bending strength of RPC significantly increases with the curing age, although notable differences in the growth rate and final strength values are observed among the various mixture ratios. From 3 to 7 days, most of the specimens show a rapid increase in the bending strength, highlighting the substantial contribution of the early hydration reaction to the bending properties. For the test group doped with a single SCM, both FAM and SF can significantly contribute to the growth of bending strength, as shown in Fig. 9a, c, d and f, the increase in bending strength can reach 2.42 MPa by FAM and 2.74 MPa by SF, with the increase of 28.54% and 29.91%, respectively. As shown in Fig. 9b, e, the test groups incorporating FAM and SF composites (0.18-F0.5-S0.5 and 0.16-F0.5-S0.5) demonstrate greater early-age strength gains, with increases of 32.4% and 31.7%, respectively. From 7 to 28 days, the bending strength continued to increase while maintaining a high level of growth. Specimens with lower water-cement ratios 0.16-F0-S1 and 0.16-F0.5-S0.5 (Fig. 9d, e) showed the highest bending strength at 28 days, showing a significant improvement compared to the specimen with a water-cement ratio of 0.2 (Fig. 9g).

##### Effects of the water-cement ratio and SCMs composition on the bending strength

**Fig. 10:**
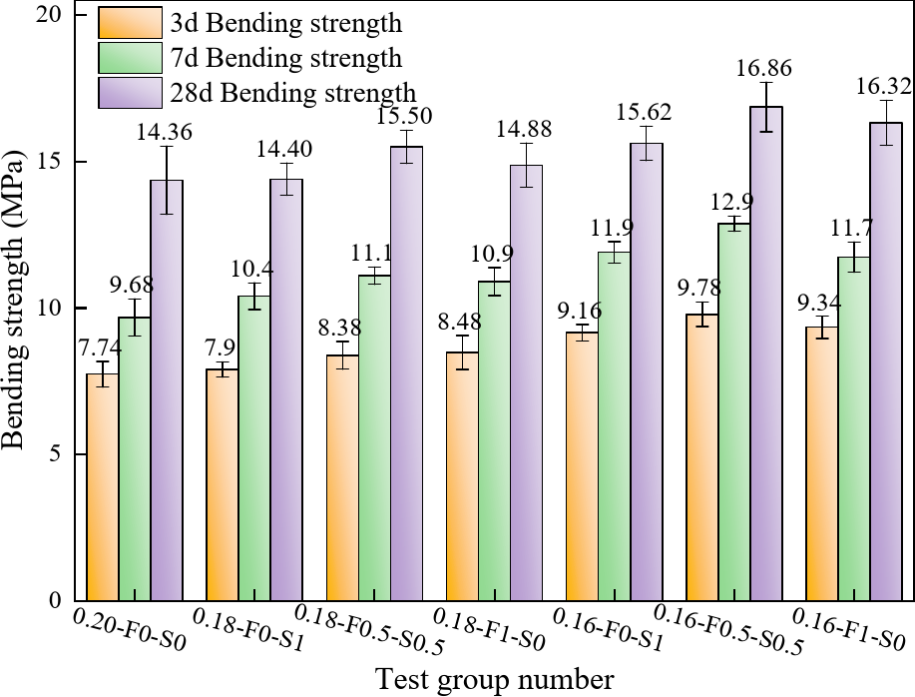
Effects of the water-cement ratio and SCMs composition on the bending strength.

Figure 10 illustrates the variation in bending strength with age for the different test groups. Overall, the bending strength of all groups increases with age, although the rate of increase and final strength are influenced by the water-cement ratio and SCMs composition. Specifically, test groups with lower water-cement ratios, such as 0.16-F0.5-S0.5, exhibit faster strength development and higher final bending strengths compared to those with higher water-cement ratios. For instance, the 0.16-F0.5-S0.5 group achieves a bending strength of 9.78 MPa at 3 days, which increases to 12.88 MPa at 7 days and reaches 16.86 MPa at 28 days. In contrast, the 0.18-F0.5-S0.5 group shows slightly lower bending strengths at the same ages, while the 0.20-F0-S0 group, with the highest water-cement ratio, exhibits the lowest bending strengths of 7.74 MPa at 3 days, 9.68 MPa at 7 days, and 14.36 MPa at 28 days. These results indicate that reducing the water-cement ratio significantly enhances the densification of the cement matrix, leading to improved bending strength.

The rate of bending strength increase varies across the test groups. For example, between 3 and 7 days, the 0.16-F0.5-S0.5 group shows a strength increase of 3.12 MPa, whereas the 0.20-F0-S0 group exhibits a smaller increase of only 1.94 MPa. This suggests that a lower water-cement ratio, combined with an optimal SCM composition, significantly enhances early strength development. Between 7 and 28 days, the strength growth rate slows across all groups, but those containing FAM (e.g., 0.18-F1-S0 and 0.16-F0.5-S0.5) continue to demonstrate substantial increases of 4.4 MPa and 3.96 MPa, respectively. This late-age strength enhancement can be attributed to the denser matrix formed in specimens with lower water-cement ratios, which promotes a more uniform fiber distribution and accelerates early strength development. Additionally, the filling effect of FAM and the pozzolanic reaction of SF increase matrix density and contribute to additional C-S-H gel formation through secondary hydration reactions, further improving strength at later ages.

## Impact resistance

### Impact damage characteristics

**Fig. 11:**
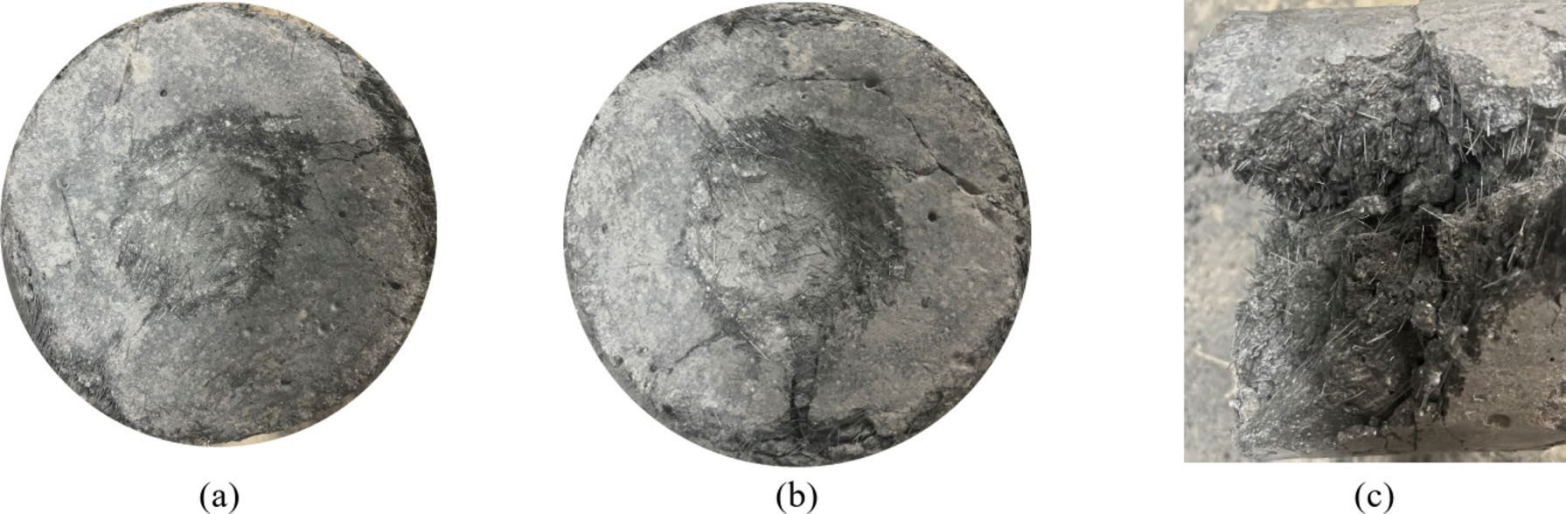
Characteristics of the impact damage caused by a drop hammer (**a**) Initial cracking (**b**) Crack development (**c**) Impact damage.

Figure 11 shows the progressive damage stages of RPC under drop hammer impact, including the incipient cracking, crack development, and impact damage stages. These stages reflect the damage progression and ultimate failure mode of the material under the accumulated impact energy.

In the incipient cracking stage, small cracks appear on the surface of the specimen, which are typically caused by the impact stress exceeding the elastic limit of the material. At this stage, the cracks, with shallow depths and a sparse distribution, are concentrated near the impact site (Fig. 11a). The initial cracks signify the onset of irreversible damage, although the material has not yet experienced large-scale failure.

In the crack development stage, the number and depth of cracks increase, with a more pronounced extension of the cracks surrounding the impact site (Fig. 11b). As the impact energy continues to accumulate, more microscopic cracks form within the material, and the crack propagation direction and density become more complex. The accelerated crack expansion in this stage indicates that the material begins to lose its structural integrity and is progressing towards failure. This stage reflects the toughness and ductility of the material, as it can still absorb impact energy while undergoing internal damage.

In the impact damage stage, the material experiences complete destruction under the final impact energy, resulting in pronounced structural cracking and fibre pullout (Fig. 11c). At this point, the material structure has been severely destabilized, and a large damaged region has formed. The visible fibre pullout and dispersion indicate that the reinforcing fibres inside the concrete have been stretched and detached under the stress, demonstrating their contribution to enhancing the material impact toughness.

#### Cracking impact number

**Fig. 12:**
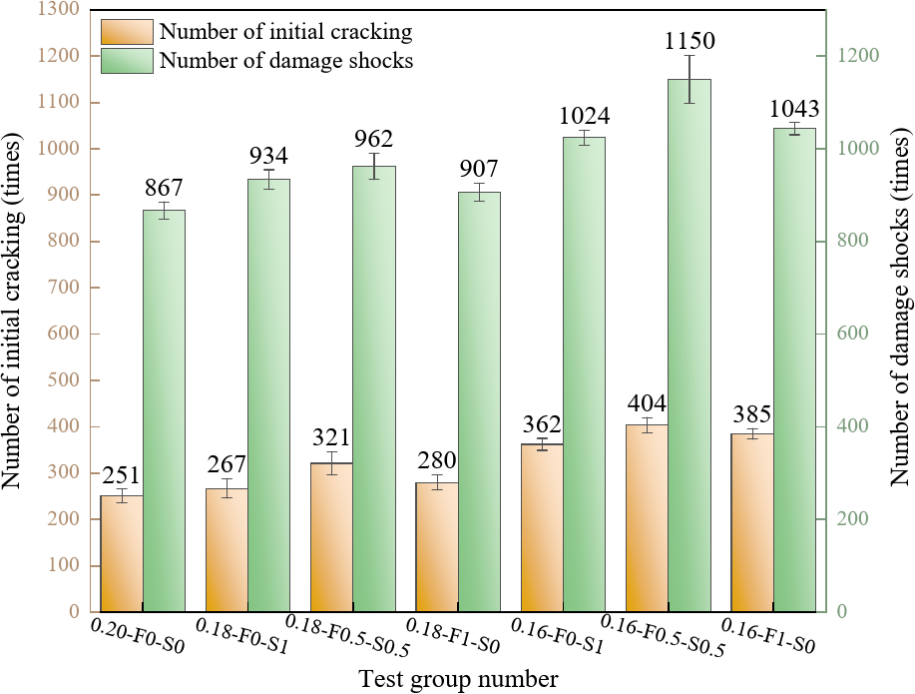
Change rules of the initial cracking and destructive impact numbers.

Figure 12 presents the initial cracking impact number and destructive impact number for the different test groups, reflecting the impact toughness of each specimen. The results clearly show that the destructive impact number increases significantly as the water-cement ratio decreases. The 0.16-F0.5-S0.5 group exhibits the highest destructive impact number of 1150, which is 19.54% and 32.64% greater than those of the 0.18-F0.5-S0.5 (962) and 0.20-F0-S0 (867) groups, respectively. This demonstrates that reducing the water-cement ratio results in a more compact matrix structure, enhancing impact resistance and prolonging the material’s ability to withstand impact loads. Furthermore, the 0.16-F0.5-S0.5 group shows a 26.8% increase in destructive impact number compared to groups containing only FAM or SF, indicating that the synergistic combination of these materials reduces porosity and densifies the cement matrix. The incorporation of microfine copper-plated steel fibers also plays a crucial role in toughening the material during crack propagation, hindering further crack expansion and enabling the specimen to endure more impacts before failure.

#### Impact resistance energy

**Fig. 13:**
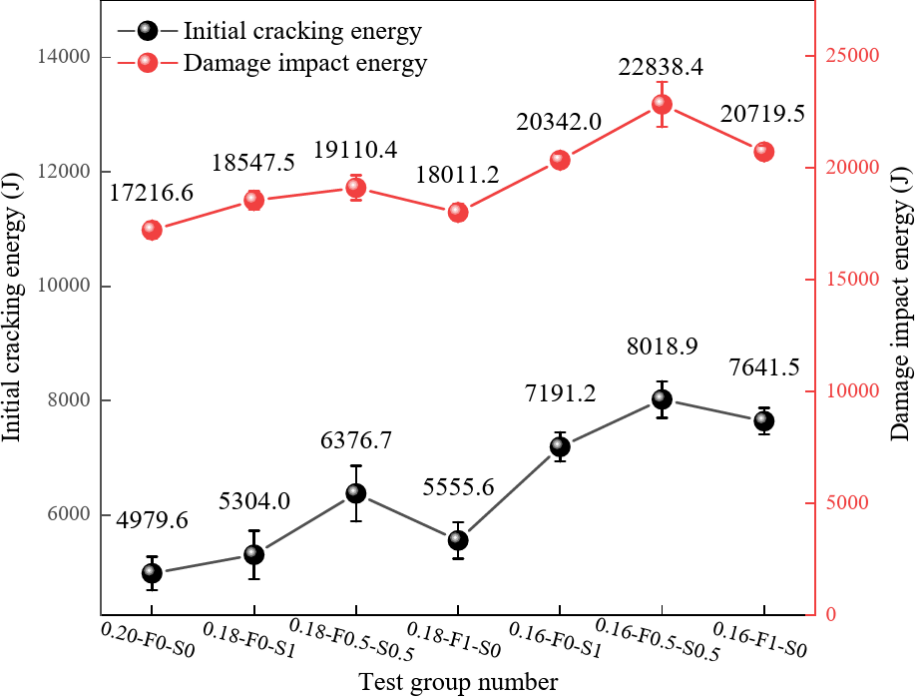
Variation patterns of the impact energies.

The initial cracking energy of the specimens exhibits a decreasing trend with increasing water-cement ratio, as shown in Fig. 13. The lowest initial cracking energy of 4979.6 J is observed for the 0.20-F0-S0 group. As the water-cement ratio decreases to 0.18, the initial cracking energy increases to 6376.7 J for the 0.18-F0.5-S0.5 group, reaching a maximum of 8018.9 J for the 0.16-F0.5-S0.5 group. These findings indicate that lower water-cement ratios enhance the densification of RPC, reduce porosity, and improve resistance to initial cracking under impact.

The SCMs combination also significantly affects the initial cracking energy. The highest initial cracking energy of 6376.7 J is observed in the 0.18-F0.5-S0.5 group, which includes 50% FAM and 50% SF. This energy further increases to 8018.9 J in the 0.16-F0.5-S0.5 group, highlighting the enhanced impact resistance achieved through the synergistic incorporation of both materials. In contrast, groups containing either FAM or SF alone exhibit lower initial cracking energy.

Regarding destructive impact energy, the values remain consistently high with adjustments to the water-cement ratio and admixture proportions. The 0.16-F0.5-S0.5 group achieves the highest destructive impact energy of 22,838.4 J, indicating superior energy absorption capacity under impact. In comparison, the 0.20-F0-S0 group exhibits a relatively low destructive impact energy of 17,216.6 J. A significant increase in destructive impact energy is observed when the water-cement ratio is reduced from 0.20 to 0.18, reaching 19,110.4 J in the 0.18-F0.5-S0.5 group. While the destructive impact energy fluctuates at a water-cement ratio of 0.16, it remains consistently high, demonstrating that lower water-cement ratios significantly enhance the destructive impact resistance of concrete.

The combination of a low water-cement ratio (0.16 and 0.18) with 50% FAM and 50% SF improves both initial cracking energy and destructive impact energy, underscoring that adjusting the water-cement ratio and incorporating SCMs can significantly increase the impact toughness of RPC. Notably, the destructive impact energy is generally greater than the initial cracking energy for the same mixture ratio, with similar trends observed for both energy types. The smaller increase in destructive impact energy suggests that material toughness plays a more significant role in energy absorption during impact damage.

#### Weibull distribution model for the impact resistance

Under identical test conditions, the variability of materials such as fibres, cementitious materials, and aggregates introduces a certain degree of dispersion in the number of impacts that RPC can withstand. As such, studying the probability distribution of this dispersion provides valuable insight into the impact resistance behaviour of a material. The impact resistance of concrete can be modelled as a fatigue damage mechanism, in which the fatigue life data are commonly described using a normal, lognormal, or Weibull distribution model. Among these models, the Weibull distribution is particularly suitable for analysing the impact resistance of concrete because of its minimal sample size requirements and simpler mathematical processing^38,39^. The Weibull probability density function for the number of impacts N can be expressed as:

4 $$\:f\left(N\right)={\frac{b}{{N}_{a}-{N}_{0}}\left(\frac{N-{N}_{0}}{{N}_{a}-{N}_{0}}\right)}^{b-1}exp\left[{-\left(\frac{N-{N}_{0}}{{N}_{a}-{N}_{0}}\right)}^{b}\right]$$

where $\:{N}_{0}$ is the minimum life parameter, with $\:{N}_{0}\le\:N<{\infty\:}$; $\:{N}_{a}$ is the characteristic life parameter; and b is the Weibull shape parameter.

When $\:{N}_{0}$=0, Eq. (4) reduces to a two-parameter Weibull distribution with a probability density function of

5 $$\:f\left(N\right)={\frac{b}{{N}_{a}}\left(\frac{N}{{N}_{a}}\right)}^{b-1}exp\left[{-\left(\frac{N}{{N}_{a}}\right)}^{b}\right]$$

where $\:P\left(N\right)$ denotes the cumulative failure probability function of the specimen against the number of impacts N. Then,

6 $$\:P\left(N\right)=1-exp\left[{-\left(\frac{N}{{N}_{a}}\right)}^{b}\right]$$

Using equivalent variations of Eq. (6) and taking the natural logarithm twice at the same time yield

7 $$\:\text{l}\text{n}\left[\text{l}\text{n}\right(\frac{1}{1-P\left(N\right)}\left)\right]=blnN-bln{N}_{a}$$

Let $\:y=\text{l}\text{n}\left[\text{l}\text{n}\right(\frac{1}{1-P\left(N\right)}\left)\right]$, $\:x=lnN$, $\:\alpha\:=b$, and $\:\beta\:=bln{N}_{a}$; then, Eq. (7) can be expressed as

8 $$\:y=\alpha\:x-\beta\:$$

The cumulative failure probability function for the small sample size condition (*N* ≤ 20), where the groups of data are arranged in ascending order and the mean rank method is used to obtain the expectation estimate, is expressed as follows:

9 $$\:P\left(N\right)=\frac{i}{n+1}$$

where i is the serial number of the damaged specimen in ascending order, with i = 1, 2, …, n.

To further assess the consistency of the adopted Weibull distribution fitting results with the experimental data, the Kolmogorov-Smirnov (KS) was used to perform the goodness-of-fit test^40^, where in Eq. (11), $\:{D}_{n}$ is the maximum difference between the Weibull distribution function of the impact resistance, $\:P\left(N\right)$ (Eq. 6) and $\:{P}_{n}\left({N}_{i}\right)$ (Eq. 10), and $\:{D}_{n,\:\alpha\:}$ is the critical value of 0.708 for the three data at 95% assurance.

10 $$\:{P}_{n}\left({N}_{i}\right)=(i-0.5)/N$$

11 $$\:{D}_{n}=\text{max}\left[\left|P\left({N}_{i}\right)-{P}_{n}\left({N}_{i}\right)\right|\right]<{D}_{n,\:\alpha\:}$$

The results of the fitting analysis of the number of impacts calculated according to Eqs. (7)-(9) are shown in Table 3. As shown in Table 3, the minimum value of the linear regression correlation coefficient R^2^ for the initial cracking impact number of RPC N_1_ is 0.9115, whereas the maximum value is 0.9998, and the minimum value of the linear regression correlation coefficient R^2^ for the destructive impact number N_2_ is 0.9303, whereas the maximum value is 0.9998. When the correlation coefficient R^2^ ≥ 0.7, the reliability of the model can be considered to meet the requirements; thus, the initial cracking impact number and destructive impact number of RPC obey the two-parameter Weibull distribution model.

The fitting analysis results for the number of impacts, calculated via Eqs. (7)-(9), are presented in Table 3. Table 3 shows that the linear regression correlation coefficient R^2^ for the initial cracking impact number N_1_ ranges from 0.9115 to 0.9998, whereas for the destructive impact number N_2_, it ranges from 0.9303 to 0.9998. When the correlation coefficient R^2^ is greater than or equal to 0.7, the model is deemed reliable. Therefore, both the initial cracking impact number and the destructive impact number of RPC follow the two-parameter Weibull distribution model. The results of the K-S fitting goodness-of-fit tests are shown in Table 3, where $\:{D}_{n}$ for the initial cracking number N_1_ and the final damage number N_**2**_ under different fitment ratios are smaller than the critical value $\:{D}_{n,\:\alpha\:}$, which further indicates that the impact resistance numbers N_1_ and N_**2**_ can obey the Weibull distribution better.

**Table 3:** Two-parameter regression of the Weibull distribution model.

Specimen number	α		β		R^2^		K-S goodness of test		
	N_1_	N_2_	N_1_	N_2_	N_1_	N_2_	N_1-_$\:{\mathbf{D}}_{\mathbf{n}}$	N_2-_$\:{\mathbf{D}}_{\mathbf{n}}$	$\:{\mathbf{D}}_{\mathbf{n},\:\varvec{\upalpha\:}}$
0.2-F0-S0	12.944	71.916	36.583	247.9	0.9288	0.9978	0.495	0.552	0.708
0.18-F1-S0	9.36	52.705	34.942	239.39	0.925	0.9998	0.559	0.530	
0.18-F0.5-S0.5	9.619	55.929	26.975	185.71	0.9115	0.9995	0.558	0.498	
0.18-F1-S0	13.671	77.433	36.932	251.92	0.9996	0.9923	0.470	0.547	
0.16-F0-S1	21.685	128.18	50.021	347.14	0.9315	0.9981	0.618	0.605	
0.16-F0.5-S0.5	19.769	119.04	17.686	125.05	0.9998	0.9998	0.502	0.489	
0.16-F1-S0	25.783	153.89	59.745	415.64	0.9662	0.9303	0.583	0.567	

According to Eq. (6) to (9), the number of impacts N under different failure probabilities can be calculated as shown in Eq. (12).

12 $$\:N=\text{e}\text{x}\text{p}\left\{\frac{\text{ln}\left[-\text{l}\text{n}\left(1-P\left(N\right)\right)+\beta\:\right]}{\alpha\:}\right\}$$

where α and β are the regression parameters of the Weibull distribution. The exact values for these parameters are provided in Table 3.

Based on Eq. (10), the numbers of impacts for different failure probabilities of RPC were calculated, as shown in Table 4. For the same material combination, the number of impacts progressively increases with increasing failure probability. For example, the damage impact number for specimen 0.16-F0.5-S0.5 is 1097 at a 25% probability of failure, and it increases to 1199 at a 75% probability of failure. This finding indicates that the ability of the specimen to withstand a greater number of impacts improves as the failure probability increases. Furthermore, the impact resistance of different material combinations follows a consistent pattern under varying failure probability, further validating the effectiveness of material modification in enhancing the impact resistance.

**Table 4:** Number of impacts for different failure probabilities.

Specimen number	25%		50%		75%	
	*N*_1_	*N*_2_	*N*_1_	*N*_2_	*N*_1_	*N*_2_
0.2-F0-S0	235	848	252	868	265	885
0.18-F1-S0	244	912	268	935	289	954
0.18-F0.5-S0.5	294	933	323	964	347	989
0.18-F1-S0	263	887	281	908	295	925
0.16-F0-S1	348	1007	363	1025	375	1039
0.16-F0.5-S0.5	387	1097	405	1153	419	1199
0.16-F1-S0	373	1029	385	1044	396	1056

## Conclusion

This study investigated the influence of the water-cement ratio and the supplementation of cementitious materials (FAM and SF) on the mechanical properties and impact resistance of RPC. The key conclusions drawn from the study are as follows:

(1) Lowering the water-cement ratio improved the compactness and reduced the porosity of RPC, resulting in increased compressive strength, bending strength, and impact resistance. The specimen with a water-cement ratio of 0.16 exhibited the best overall mechanical performance, with a compressive strength of 134.4 MPa, a bending strength of 16.86 MPa, and the ability to withstand 1150 destructive impacts.

(2) The combined use of FAM and SF significantly improved the mechanical properties of RPC. The microfine structure of the FAM helped fill the pores in the concrete, thus increasing its densification, whereas the SF promoted the cement hydration reaction through the volcanic ash effect, further enhancing the strength and durability of the material. The optimal mechanical properties were achieved when FAM and SF each accounted for half of the SCMs content.

(3) The combination of a low water-cement ratio and an appropriate amount of SCMs improved not only the compressive and bending strengths of RPC but also its impact resistance. The optimal mixture (0.16-F0.5-S0.5) demonstrated a high energy absorption capacity at initial cracking (8018.9 J) and a remarkable energy absorption capacity of 22838.4 J at the damage stage, significantly outperforming the other ratios. The distribution of the impact resistance was analysed using the Weibull distribution model, and the results confirmed that the synergistic effect of a low water-cement ratio and the inclusion of SCMs substantially improved the impact toughness of the material.

## Data Availability

Data are contained within the article.
